# Medical Men and Midwives

**Published:** 1910-04-16

**Authors:** Beatrice Webb

**Affiliations:** 41 Grosvenor Road, S.W.


### Medical Letter-Box.

### MEDICAL MEN AND MIDWIVES.

*To the Editor of* The Hospital.

Sir,—Will you allow me to call attention to a proposed grave injustice to thousands of poor women? At present, when a woman in her hour of trial is attended by a midwife, the midwife is required by statute to summon a doctor if any complication ensues. No provision is at present made for the payment of the doctor whom the State, not the patient, insists on summoning. This is an injustice to the doctor.

Now the Government proposes (in the Bill just introduced by Lord Wolverhampton) to require the Board of Guardians to pay the fee (as parochial relief to the woman and her husband). This is a grave injustice to these poor families. The woman and her husband are not paupers; they are not even destitute persons. Through the exercise of thrift they have made for themselves the normal provision for childbirth of their class, *i.e.* a midwife. The Government proposes to make them compulsorily into paupers (even if they subsequently repay the full amount it makes no difference in this respect); their homes will be visited by the relieving officer with his hated inquiries, often to the detriment (as any nurse or midwife will testify) of the woman's health; they will be liable to be proceeded against by the Board of Guardians, if, as is intended by Clause 17, sub-clause 2, the "relief" is given "on loan," and compelled to repay a charge which they have never incurred, but which the State, in the public interest, has chosen to require.

I cannot believe that the House of Lords will choose this moment to thrust thousands of poor women involuntarily into pauperism, or that the House of Commons will think of tolerating such an injustice.

When the police call a doctor to attend to a patient in an emergency, the fee is paid out of the municipal funds, and the patient is not thereby made a pauper. The Town Councils of Manchester and Liverpool are already, with the knowledge and consent of the Local Government Board, following a similar course with regard to the doctors called in by the midwives; and this course is open to any other sanitary authority (under Section 133 of the Public Health Act). There is accordingly no need for the degrading and insulting Clause 17 of Lord Wolverhampton's Bill, and I hope that the Government will withdraw it.

I am, &c.,

41 Grosvenor Road, S.W., April 1910. Beatrice Webb.

